# Neural representation of human experimenters in the bat hippocampus

**DOI:** 10.1038/s41593-024-01690-8

**Published:** 2024-07-02

**Authors:** Madeleine C. Snyder, Kevin K. Qi, Michael M. Yartsev

**Affiliations:** 1 Department of Bioengineering, UC Berkeley, Berkeley, CA USA; 2 Biophysics Graduate Group, UC Berkeley, Berkeley, CA USA; 3 Helen Wills Neuroscience Institute, UC Berkeley, Berkeley, CA USA

**Keywords:** Learning and memory, Neuronal physiology

## Abstract

Here we conducted wireless electrophysiological recording of hippocampal neurons from Egyptian fruit bats in the presence of human experimenters. In flying bats, many neurons modulated their activity depending on the identity of the human at the landing target. In stationary bats, many neurons carried significant spatial information about the position and identity of humans traversing the environment. Our results reveal that hippocampal activity is robustly modulated by the presence, movement and identity of human experimenters.

## Abstract (web summary)

Snyder et al. report that hippocampal neurons in Egyptian fruit bats modulate their activity depending on the position and identity of human experimenters when bats are flying and encode experimenter position and identity when bats are stationary.

## Main

Human experimenters are commonly present in laboratory environments, where they move and interact with subject animals during experiments. Yet, the behavior of humans in such settings is rarely monitored or reported. Studies have shown that the presence, actions and sex of humans can influence the animal’s behavior^1–5^, as well as volume-averaged and time-averaged neural responses such as local field potentials^2^ or immediate early gene expression^6^. However, the impact of human experimenters on the neural dynamics of single neurons in behaving animals remains entirely unknown. To address this, we focused on the dorsal hippocampus, a region known to encode positional information and environmental factors^7–9^, and the study of which often involves humans actively interacting with the animal subjects^7,10–18^. We explicitly tested whether and how neural dynamics of hippocampal neurons are influenced by the presence and actions of human experimenters. We chose to use the Egyptian fruit bat, whose highly structured spatial behavior^19^ affords a rigorous control over behavioral variability. This allowed us to disentangle the ongoing neural modulation related to the presence and behavior of human experimenters from the positional coding prevalent in the hippocampus.

## Stable flight behavior across experimenters during a reward task

**Fig. 1:**
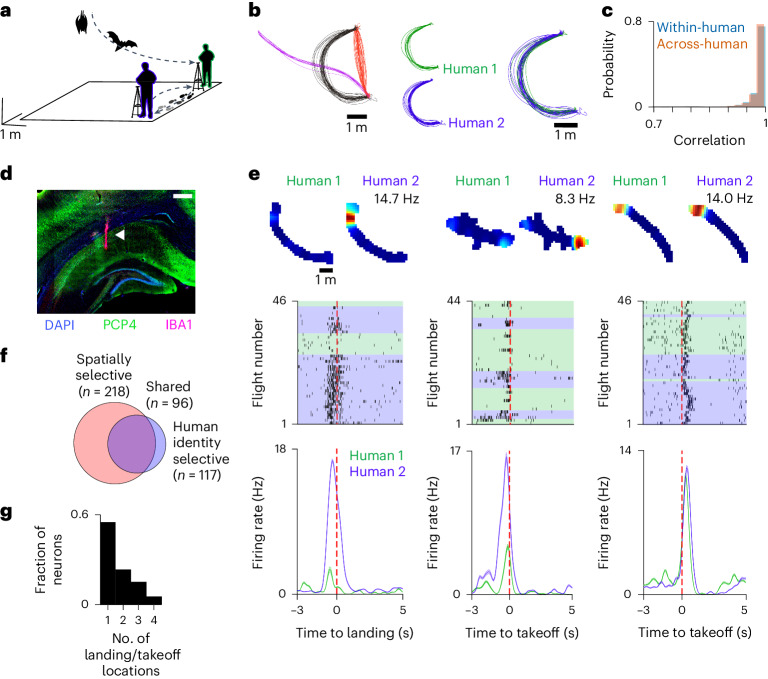
Hippocampal activity is modulated by experimenter identity during flight. **a**, Schematic of the experimental setup. Two bats flew for a reward in a self-paced task in which two human experimenters (green and blue outline) provided the reward at different fixed locations around the room. Only two tripods are shown for illustrative purposes; bats and humans are not to scale (Methods). Scale bar relates to room dimensions. **b**, Left: top view of the three most executed trajectories during a representative session. Colors denote different flight trajectories of one bat. Middle: example trajectory with flights denoted by the identity of the human, experimenter 1 (green) or experimenter 2 (blue), at the landing target. Right: flight trajectories are overlaid. **c**, Histogram of correlation values between flights of the same trajectory to the same (light blue) or different (orange) humans for all bats and all sessions (*n* = 18,450 within-human flight pairs, *n* = 14,572 across-human flight pairs). **d**, Coronal section of the dorsal hippocampus from one recorded bat, stained for DAPI, PCP4 and IBA1. A total of 14 out of 16 tetrodes (across four microdrives and four bats) were successfully identified and localized in the dorsal hippocampus. White arrowhead denotes tetrode tracks. Scale bar, 500 μm. **e**, Three representative units, with the left and middle units showing modulation of activity depending on the identity of the human at landing and a third one (right) that does not. First row shows 2D rate maps for all flights from (to) the same location, grouped by the identity of the human at take-off (landing). Peak firing rate is indicated. Second row shows the raster plot of that same neuron for all flights included in the 2D rate map. Background color corresponds to the identity of the experimenter at landing (green is human 1; blue is human 2). Third row shows the peri-stimulus time histogram (PSTH) of the raster plot above. Color of PSTH matches the color in the raster. Shaded area in PSTH denotes s.e.m. **f**, Number of units that carried significant spatial information about the bat’s position during flight (red), and those that significantly modulated their firing rate at take-off and/or landing according to the identity of the human at landing (blue). **g**, Number of landing and/or take-off locations for which a neuron was significantly modulated depending on the identity of the human at landing. Only neurons that could be analyzed at four or more locations were included in the analysis (*n* = 134 cells). Source data

**Extended Data Fig. 1:**
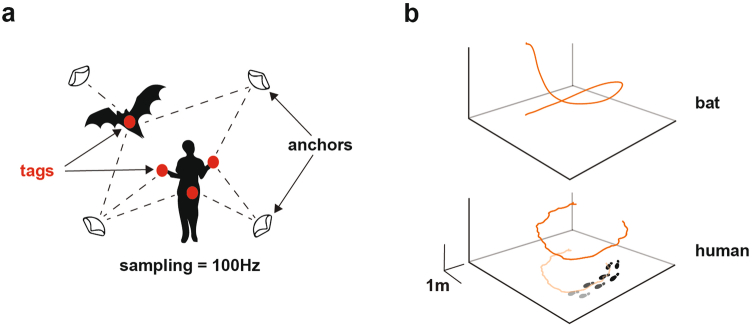
Real-Time-Location-System for tracking the positions of bats and humans, and behavioral stability. **a**. Schematic of the Real-Time-Location-System. A set of static anchors communicate with a wireless tag (red dots) for 3D position localization. A total of eight tags are used in the experiment: one tag per bat, two tags are attached to the wrists of the two humans, and one tag is carried in each of the humans’ right coat pockets. **b**. Example flight executed by a bat (top) and example traverse executed by a human (bottom) tracked with the RTLS system (Methods). For the human, a projection on the floor is also shown.

**Extended Data Fig. 2:**
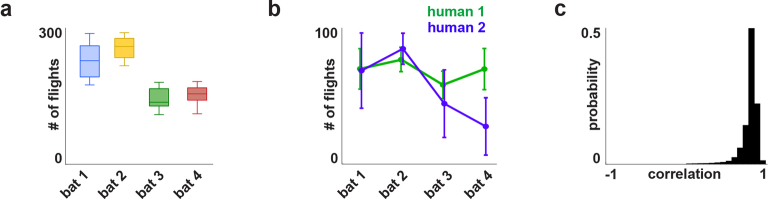
Stability and reproducibility of bat flight behavior. **a**. Distribution of the total number of flights executed by each bat over all analyzable recording sessions (n = 31 sessions). Box centers indicate the median number of flights, box bounds indicate 25th and 75th percentiles, error bars extend to minima and maxima. **b**. Average number of flights to human #1 (green) and human #2 (blue) for each bat over all analyzable recording sessions (n = 31 sessions). Error bars indicate standard deviation. **c**. Pearson correlation of the velocity profiles (concatenated x, y, and z velocity) between all pairs of flights of the same trajectory with sufficient flights to each human, across all analyzable sessions and bats (n = 37,521 flight pairs).

To examine the influence of human experimenters on neural activity in the bat hippocampus, we designed a spatial reward task in which pairs of bats could spontaneously fly to two experimenters standing at different locations in a room to obtain a fruit reward from their hand (Fig. 1a and Methods). To minimize variability, the experimenters’ hands were rested on fixed platforms of identical heights and were covered by gloves of the same size (Methods). Every 5 min, the experimenters swapped locations with one another to allow sufficient sampling of human positions and flight behavior. To monitor the spatial behavior of both bats and humans, we used a real-time location system (RTLS) that recorded the three-dimensional (3D) position of both bats and humans in the room simultaneously with high spatiotemporal precision^20^ (Extended Data Fig. 1). The bats were very active during the behavioral sessions, resulting in many flights landing at the same locations but on different human experimenters (217 ± 59 total flights per session per bat, mean ± s.d., *n* = 4 bats; Extended Data Fig. 2a,b). Importantly, the bats spontaneously flew highly structured flight paths that were repeated at high precision towards different experimenters (Fig. 1b,c and Extended Data Fig. 2c). This provided a natural ‘behavioral clamp’ on the bats’ spatial variability across flights, which in turn allowed rigorous assessment of modulation in the neural activity associated with a particular human experimenter at the landing location.

## Hippocampal neurons are modulated by experimenter identity in flight

**Extended Data Fig. 3:**
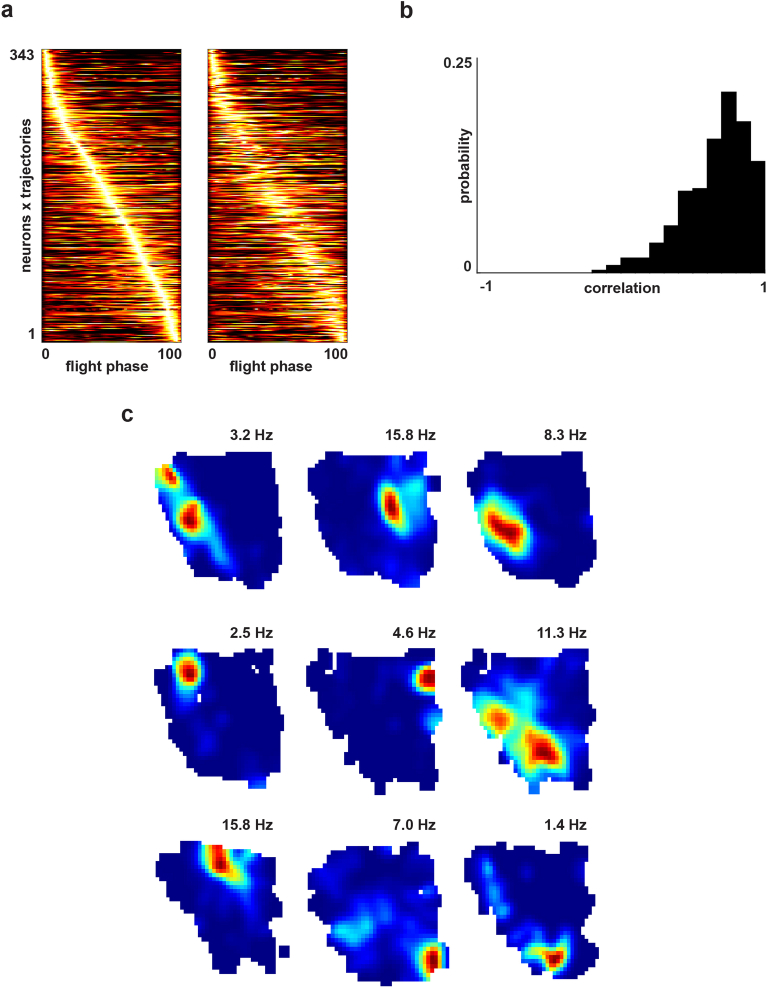
Stability and selectivity of firing fields. **a**. Left: Peak-normalized 1D rate maps for all spatially significant neurons x trajectories (even trials) sorted by peak activity over time (n = 343 neurons x trajectories). Right: 1D rate maps of all spatially significant neurons x trajectories (odd trials), sorted according to the even trials. **b**. Distribution of Spearman correlations between pairs of 1D rate maps constructed from even and odd trials, for each neuron x trajectory (n = 343 neuron x trajectory pairs). **c**. Representative 2D rate maps of significantly spatially selective neurons. Peak firing rate is indicated.

**Extended Data Fig. 4:**
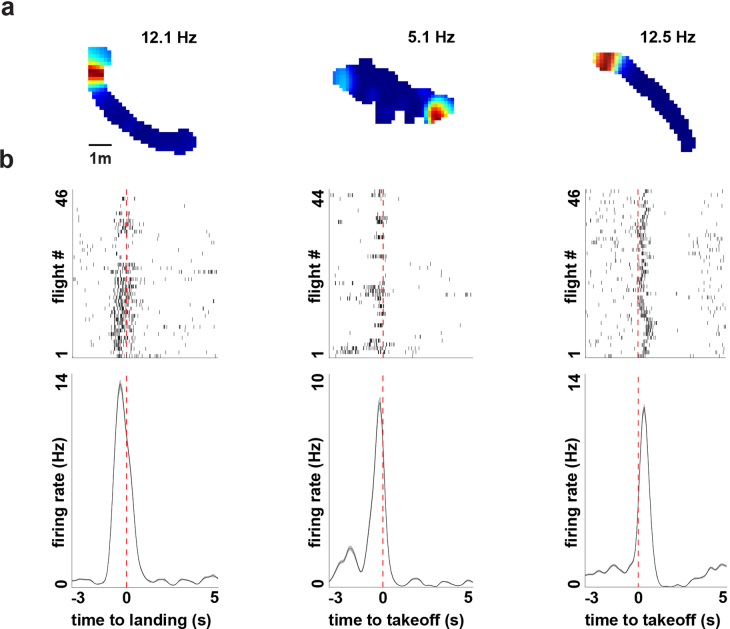
Representative examples of trial-to-trial variability in the firing of spatially selective neurons. **a**. Three example units. Same units as in Fig. 1d. First row shows 2D rate map constructed from all flights that share the same landing/takeoff location. Peak firing rate indicated**. b**. Raster plots (top) and PSTH (bottom) for all flights of that landing/takeoff location, aligned to landing (left), takeoff (middle), and takeoff (right) (red dotted line) and sorted by time of execution in the session. Shaded area of PSTH indicates standard error of the mean.

**Extended Data Fig. 5:**
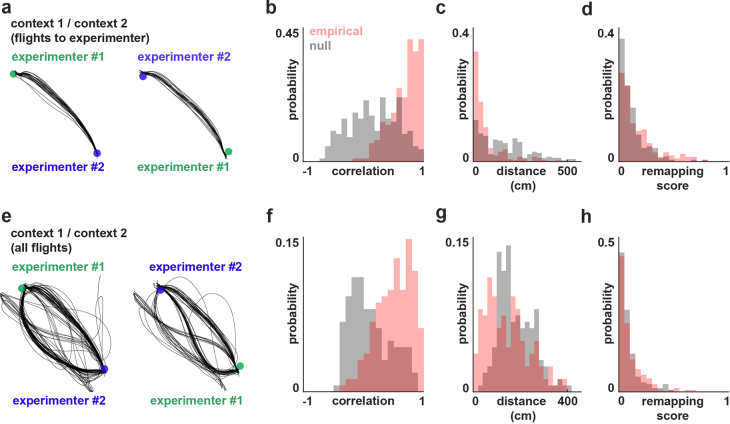
Rate remapping on 1D and 2D rate maps. **a**. Example flights along a single trajectory, during a single session, to experimenter #1 (shown in green; dot indicates mean human position during included flights) and experimenter #2 (shown in blue). **b**. Distribution of Pearson correlations between 1D linearized rate maps of trajectories to different human landing targets (n = 219 neurons x trajectories). Significant difference between empirical (red) and null (grey) distributions (*p* = 6.0 × 10^−30^, two-sided Wilcoxon signed rank test). **c**. Distribution of distance between peaks of 1D linearized rate maps of trajectories to different human landing targets (same neurons x trajectories as in subpanel ‘**b**’). Significant difference between empirical (red) and null (grey) distributions (*p* = 1.2 × 10^−15^, two-sided Wilcoxon signed rank test). **d**. Distribution of remapping scores for 1D linearized rate maps of trajectories to different human landing targets (same units x trajectories as in subpanel ‘**b**’). Significant difference between empirical (red) and null (grey) distributions (*p* = 0.002, two-sided Wilcoxon signed rank test). e. Example session of flights included in 2D rate maps for classical rate remapping analysis (included are all flights in a given context (Methods)). **f**. Distribution of Pearson correlations between 2D rate maps of different contexts (n = 162 neurons x contexts). Significant difference between empirical (red) and null (grey) distributions (*p* = 5.0 × 10^−^^8^, two-sided Wilcoxon signed rank test). **g**. Distribution of center of mass movements between rate maps of different contexts for same units as in subpanel ‘**f**’. Significant difference between empirical (red) and null (grey) distributions (*p* = 4.7 × 10^−5^, two-sided Wilcoxon signed rank test). **h**. Distribution of remapping scores between rate maps of different contexts for same units as in subpanel ‘**f**’. No significant difference between empirical (red) and null (grey) distributions (*p* = 0.056, two-sided Wilcoxon signed rank test).

**Extended Data Fig. 6:**
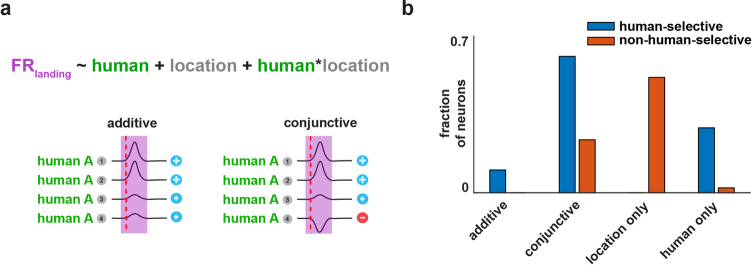
Linear model predicting firing rate at landing based on human identity, location and their interaction. **a**. Schematic of the linear model using one non-ordinal categorical variable for human identity, one nonordinal categorical variable for landing location, and their interaction term (Methods). The grey circles with numbers represent possible locations, the red dotted line indicates landing, the purple boxes indicate the period used to calculate the firing rate, and the cartoon neural traces and corresponding plus and minus signs illustrate the difference in neural modulation that results from a strictly additive code versus a conjunctive code (Methods). In this cartoon example the neuron is modulated differently at the intersection of Human A and Location 4 than would be expected in an additive code. For brevity only one example experimenter (Human A) is included in this example. **b**. Model comparison was performed to determine if a given variable significantly (model comparison using anova test; *p* < 0.05) improved the prediction of a neuron’s firing rate at landing (Methods). Neurons classified as ‘additive’ had significant Human and Location terms in model comparison, but not significant interaction terms. ‘Conjunctive’ neurons had significant Human and/or Location terms, as well as a significant interaction term. ‘Human only’ neurons had a significant Human term, but not significant Location or interaction terms. ‘Location only’ neurons had a significant Location term, but not significant Human or interaction terms. These categories of neurons were then split according to whether they modulated their activity depending on the human at takeoff/landing (via permutation test, Methods). Bar plot shows the split in the fraction of neurons for each category (n = 127 neurons). Note the fractions of additive, conjunctive, and human-only neurons that were also classified as modulated by the human (via permutation test) are larger than the fractions that were not modulated, and there are no neurons in the location-only category that were also classified as modulated by the human (via permutation test).

We wirelessly recorded the activity of 307 dorsal CA1 hippocampal neurons from four bats engaged in the behavioral task (Fig. 1d, Methods and Supplementary Fig. 1). Most of the units that were active during flight fired primarily around take-off and landing, and carried significant spatial information about the recorded bats’ position (84.2%, or 218 out of 259 flight-active cells, when assessed in 2D; 71.3%, or 176 out of 247 flight-active cells, along specific trajectories; Bonferroni corrected; Methods and Extended Data Fig. 3), consistent with an allocentric representation of self-position within the environment^7,8,21^. However, when inspecting activity of each unit across flights, we also observed substantial variability in the neural responses (Extended Data Fig. 4). We therefore asked whether the firing of hippocampal neurons might also be modulated by the spatial locations and identities of other individuals—conspecifics or humans—in the room. We found that nearly half of the neurons significantly modulated their firing rates depending on the identity of the experimenter at the landing location (48%, 117 out of 244 analyzable units; Fig. 1e,f and Extended Data Fig. 4b (left and middle examples); remapping quantified in Extended Data Fig. 5 and Supplementary Fig. 2; see also Methods). Changes in firing rates were stable between earlier and later parts of the session (Supplementary Fig. 3). Moreover, most neurons were significantly modulated by human identity in only one location (Fig. 1g and Methods), pointing to a possible conjunctive code for positional and experimenter-identity information during spatial movement. To further examine the extent to which human identity and location significantly contribute to modulation of firing upon landing, we used a simple linear model (Extended Data Fig. 6a). This model enabled us to disambiguate between neurons using an additive code versus a conjunctive coding for human identity and location. The results were in agreement with the above finding and suggested that the conjunction of human identity and location is a significant contributor to the modulation of the activity of many hippocampal neurons (*n* = 127 neurons; Extended Data Fig. 6b and Supplementary Fig. 4).

**Extended Data Fig. 7:**
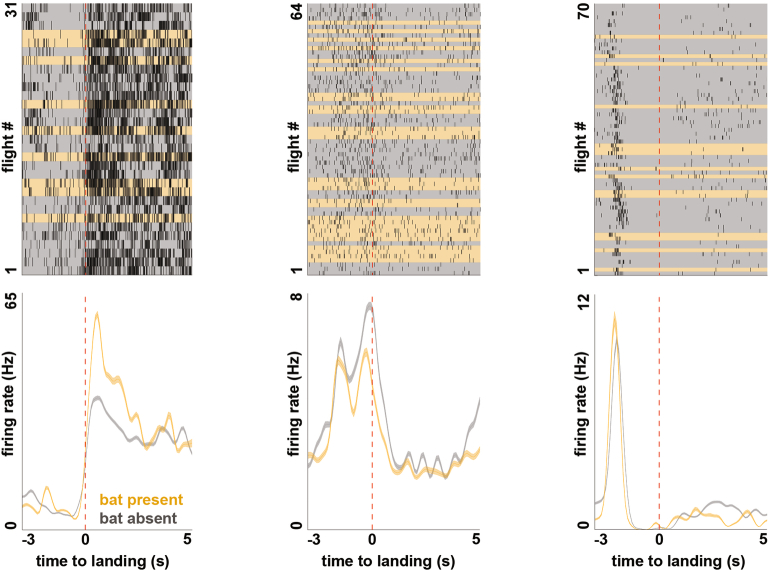
Firing rate modulation during flight by the presence of another other bat at the landing location. Shown are raster plots and PSTHs’ for three example units that modulate (or not) their activity depending on whether there is another bat present at the landing target, aligned to landing (red dotted line). Colored rows on the raster plots indicate on which flights the other bat was present (orange) or absent (grey) at the landing target. Colored lines on the PSTH plots indicate which PSTH was calculated from flights that landed on another conspecific (orange) or not (grey). Shaded lines indicate standard error of the mean.

**Extended Data Fig. 8:**
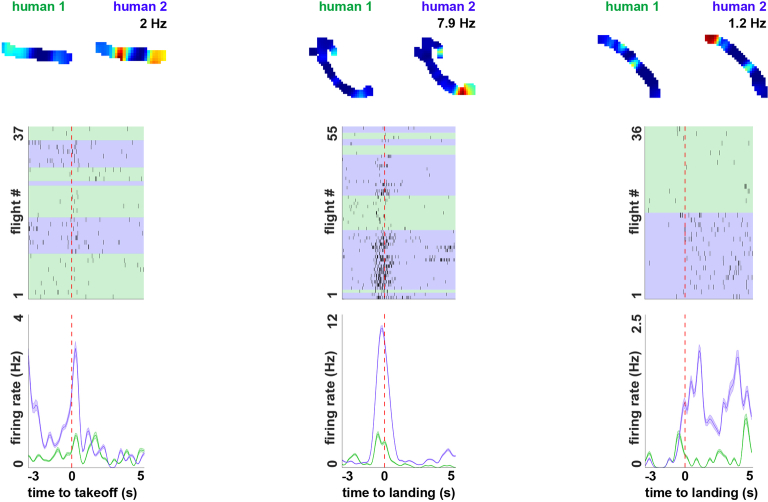
Modulation of firing by human identity in the absence of another bat at the landing target. Top row shows 2D rate maps of three example units that modulate their activity on flights to the same location but different humans. No flights included here had another bat present at landing. Second and third rows show raster plots and PSTH plots of the above units. Colored rows on the raster plots indicate which flights landed on human #1 (green) versus human #2 (blue). Colored lines on PSTH plots indicate which PSTH was calculated from flights that landed on human #1 (green) versus human #2 (blue). Shaded areas indicate standard error of the mean.

Consistent with previous reports^20^, we also found a subpopulation of neurons that were significantly modulated by the presence or absence of a conspecific at the landing location (8.4%, 16 out of 191 analyzable units; Extended Data Fig. 7). To test whether this result could account for the differences in neural responses observed during flights towards different human experimenters, we constrained our analysis to only include flights for which the other bat was not present at the take-off or landing location. Even with this additional constraint, we found that over 40% of the units (41.2%, or 77 out of 187 analyzable units) had significantly different firing activity depending on the identity of the human at the landing location (Extended Data Fig. 8). Therefore, the presence of a conspecific could not fully account for the neural modulation associated with the identity of the human experimenter. Furthermore, differences in reward quantities provided by different experimenters on a subset of the sessions (Methods) also could not fully account for changes in neural modulation (Supplementary Fig. 5). Together, these findings suggest that during self-motion, information about the identity and positions of humans in the environment explains a substantial portion of variance in hippocampal activity that cannot be accounted for by changes in self-position, movement patterns, reward quantity or the presence of conspecifics.

## Hippocampal neurons are informative of experimenter location and identity during rest

**Fig. 2:**
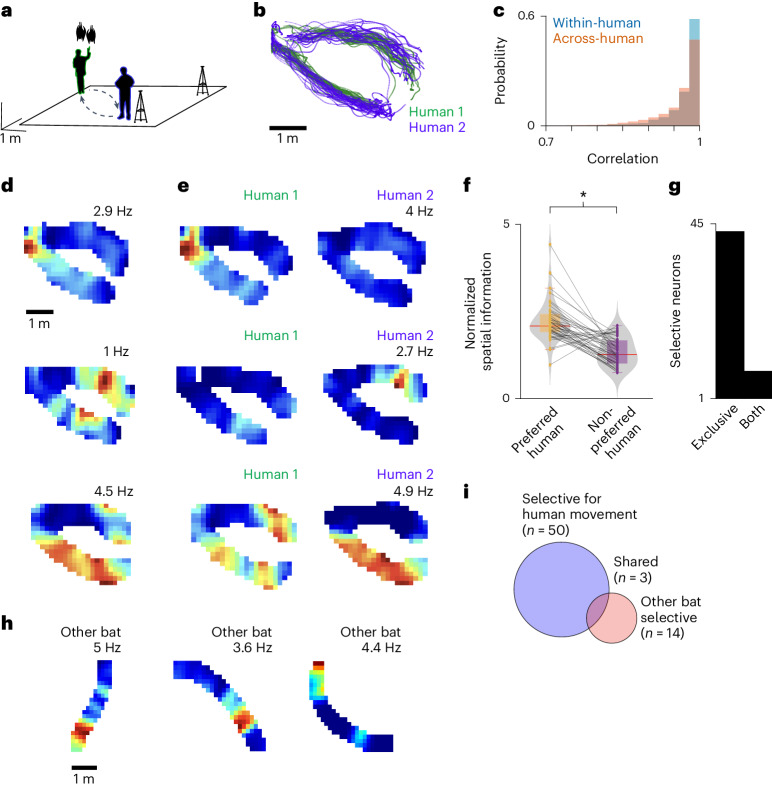
Hippocampal activity is modulated by experimenter movement and identity during rest. **a**, Schematic of the experimental setup. Two human experimenters repeatedly approached the bats hanging in their preferred resting location. **b**, Top view of tracked position of both humans (green, human 1; blue, human 2) performing traverses during a representative session. **c**, Histogram of correlation values between traverses within (light blue) and across (orange) the different human experimenters (*n* = 5,904 within-human traverses, *n* = 5,829 across-human traverses). **d**, Two-dimensional rate maps of three representative units (rows) showing significant spatial selectivity for human position while the recorded bat was stationary. Peak firing rate is indicated. **e**, Two-dimensional rate maps of the same three units in **d**, but split according to human identity. **f**, Normalized spatial information for the preferred human (for which a unit carried significant spatial information) and the non-preferred human (*n* = 43 neurons). *P = 1.1 × 10^−8^ (two-sided Wilcoxon signed-rank test); see Methods. Median indicated by thick red line on boxplot; bounds of box indicate 25th and 75th percentiles; error bars indicate minima and maxima. Violin plot shows kernel density estimate. Gray lines connect the same neuron across conditions. **g**, Numbers of neurons that carried significant spatial information for one human or for both humans (total *n* = 50 neurons). Note that nearly all neurons were selective for only one human. **h**, Two-dimensional rate maps of three representative units, showing significant spatial selectivity for the position of the other flying bat while the recorded bat was stationary. Peak firing rate is indicated. **i**, Number of units that carry significant spatial information about the location of either a human experimenter (blue) or the other bat in the room (red). Source data

**Extended Data Fig. 9:**
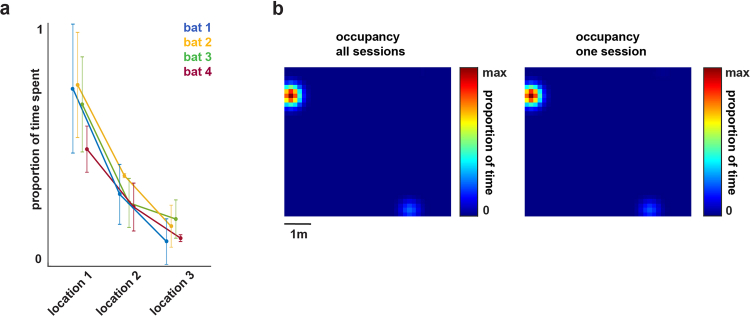
Distribution of bats’ rest locations during human traversals. **a**. Mean proportion of total roost time each bat spent at self-selected rest locations during experiment #2 (human movement while bats were stationary), ordered from most preferred rest location to least preferred rest location. Shown are top three rest locations for each bat across all analyzable sessions (n = 31 sessions), which explain an average of 84% of the roost time. Error bars indicate standard deviation. **b**. Left: occupancy (color bar) of one bat’s roosting locations across all sessions (n = 8 sessions) of experiment #2, smoothed with gaussian kernel (σ = 1.5 bins). Right: occupancy of one bat’s roosting locations across a single example session of experiment #2, smoothed with gaussian kernel (σ = 1.5 bins).

During many behavioral experiments, the human experimenter ‘performs a task’ in the presence of the animal, such as distributing food, handling the subject(s) or moving from one location to another^10–12,14–17^. We therefore conducted an additional experiment to explicitly ask whether hippocampal activity in a stationary bat contains information about the position and movement of experimenters in the room (Fig. 2a). To compare movement across experimenters while controlling for the bats’ position, we leveraged the bats’ natural tendency to rest in self-selected, yet highly consistent, locations in the room^20^ (Extended Data Fig. 9). While the bat was resting, experimenters deliberately repeated fixed traverses from a designated starting location towards the bat (Fig. 2b,c and Supplementary Fig. 6; see also Methods). At the resting location, the human dispensed the reward or briefly handled the bat and then followed a fixed path back to the start location. Experimenters randomly took turns performing traverses. When pooling all traverses from both humans, we found that a subpopulation of neurons carried significant spatial information about the position of the experimenter moving through the room (20.3%, 44 out of 217 analyzable units; Fig. 2d and Methods). Given our observation of neuronal selectivity for human identity in flying bats (Fig. 1), we asked whether similar identity selectivity would also be observed in stationary bats. We therefore split the trials according to the identity of the human traversing the environment and found that over a third of the units were significantly spatially informative about the position of at least one experimenter (36%, 50 out of 139 analyzable units; Fig. 2e). Intriguingly, most identity-responsive neurons carried significant spatial information exclusively for one experimenter (86%, 43 out of 50 analyzable units; Fig. 2f,g). Furthermore, there was little overlap in the subpopulations of neurons modulated by the identity of stationary humans during self-motion and those modulated by the position of moving humans while the bats were at rest (10.6%, 7 out of 66 analyzable units), pointing to largely independent populations selective for human identity depending on the bats’ behavioral state. Finally, we found roughly similar proportions of neurons selective for the human dispensing the reward (46%, or 23 out of 50 analyzable units) or handling the bats (40%, or 20 out of 50 analyzable units), with 14% of the units responding to both humans (7 out of 50 analyzable units).

**Extended Data Fig. 10:**
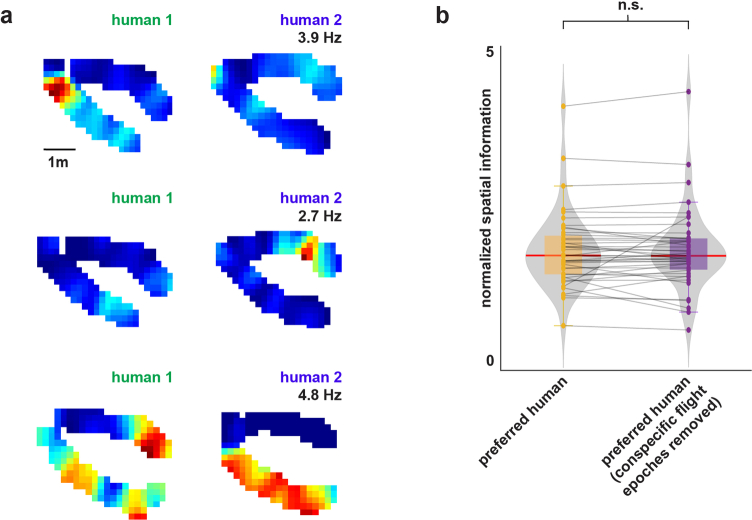
Neurons with significant spatial information for human position and identity, excluding all bouts when the other bat was flying. **a**. 2D rate maps of the same example neurons shown in Fig. 2d but excluding all times when the other bat was flying. **b**. For all neurons that carried significant spatial information for just one human, we compared the normalized spatial information (Methods) for the ‘preferred’ human (for which spatial information was significant) before (left) and after (right) removing all epochs when the conspecific bat was flying. Note that there was no significant difference (n.s.) between these two conditions (n = 43 neurons, two-sided Wilcoxon signed rank test, *p* = 0.44). Box plots indicate median normalized spatial information, box bounds indicate 25^th^ and 75^th^ percentiles, error bars extend to minima and maxima. Violin plots show kernel density estimation.

Previous reports suggested that the activity of hippocampal neurons in stationary animals may carry information about the position of a conspecific that is moving^11,22^ (but see ref. ^23^). We therefore asked whether there was a comparable population of neurons encoding information about the position of the other bat in the room, and importantly, whether this might account for our results. We found a modest population of neurons that carried significant spatial information about the position of the other conspecific while the recorded bat was at rest (Fig. 2h; 10.8%, or 14 out of 130 analyzable units). Interestingly, the populations encoding the conspecific and human position were minimally overlapping (4.7%, or 3 out of 64 analyzable units; Fig. 2i). Finally, excluding all epochs of conspecific flight during human traverses had no significant impact on the results (Wilcoxon signed-rank test, *P* = 0.44; Extended Data Fig. 10). Together, these findings suggest that there are units in the dorsal CA1 region of the hippocampus with a conjunctive code for the identity and position of the experimenter traversing the environment while the animal is stationary.

Human observation and intervention shapes animal behavior in the laboratory^1–5^. Our study asked whether experimenter presence and behavior influenced the ongoing activity of hippocampal neurons recorded from animals engaged in a spatial behavioral task. Leveraging the highly structured spatial behavior of bats, we found that neural responses were robustly modulated by the experimenters when bats were flying or stationary. These findings emphasize the potential influence of the human experimenter on the dynamics of single neurons recorded from behaving animal subjects.

In this study, we explicitly tracked the position of each human interacting with the animal subjects, but this is not the case in most experiments, even those in which the experimenters commonly interact with the animal subject during neural recordings. These interactions include delivering a reward^10,11,18^, physical relocation^12,13^ and manipulating objects or barriers as part of the behavioral task^11–15^. In these and other cases, the human is spatiotemporally coupled with the animal’s behavior in the environment. Given our findings, we encourage the neuroscience community to control, and if possible eliminate, the immediate and latent effects of experimenter behavior on neural data collected during a behavioral task.

The scope of this work was intentionally confined to a minimal number of subjects during an experimental session (two bats and two humans) while still providing sufficient complexity to assess whether the presence, behavior and identities of humans impacted ongoing neural activity. Yet, further work is needed to elucidate the complex interactions that emerge between experimenters, subjects and other salient features of the environment. Such studies should systematically vary the number of humans, animals and their relationship history while controlling for behavior. Additionally, given that this study focused on female Egyptian fruit bats, it would be important to further investigate experimenter representation across species and sensory modalities as well as across sexes of both experimental animals and researchers.

Our study focused on interspecies interactions in the laboratory environment. In the wild, most animals navigate environments populated by members of other species or live within demarcated territories, making interspecies interaction and representation a critical factor in shaping behavior and spatial decisions. The behavioral narrative of interactions between different species is often driven by the degree of overlap in their ecological niches or their relationship as recognized predator and prey. For example, interspecies relationships surrounding feeding and hunting behaviors have been characterized across taxa^24^ and include co-predation and competition for small prey between humans and reticulated pythons^25^, cooperative hunting amongst grouper fish and moray eels^26^, domestication of aphids by ants^27^ and cooperative hunting between groups of surface-hunting fish and seabirds^28^. However, very little is known about the neural landscape during interspecies interactions. In the laboratory environment, the human experimenter and the animal subject are engaged in a complex and dynamic relationship that is suitable for rigorous study of the neural basis of interspecies interactions. This line of investigation lends itself well to incorporating a diversity of animal models across a range of subdisciplines^29^ and highlights the importance of eliminating the uncontrolled effects of humans on the examined neural phenomena.

## Methods

### Subjects

Neural data were collected from four adult female Egyptian fruits bats (*Rousettus aegyptiacus*), approximately ~110–130 g in weight. All bats were housed in a humidity-controlled and temperature-controlled room. Bats implanted with a lightweight four-tetrode microdrive (Harlan 4 drive; Neuralynx) were initially singly housed in small cages, and subsequently, following recovery from surgery, co-housed in large cages. Lights in the housing room were maintained on a 12-h reverse light/dark cycle (lights off at 07:00 and lights on at 19:00). All experiments were performed at the same time of day during the bats’ waking hours (dark cycle). All experimental procedures were approved by the Institutional Animal Care and Use Committee of the University of California, Berkeley.

### Behavioral setup

All experiments were performed in a room (5.6 m × 5.2 m × 2.5 m) shielded from acoustics, electricity and radiofrequency with high-precision lighting control^30^ under uniform illumination (luminance level, 5 lux). To minimize acoustic reverberation and dampen noise from outside the room, the walls and ceiling of the flight room were covered with a thick layer of acoustic foam. A layer of acoustic absorbing black felt was placed on top of the acoustic foam, and on the floors, to prevent the bats from damaging the foam or the floors and to provide additional acoustic dampening. A layer of black netting was placed on top of the felt on the walls for the bats to hang. In addition to the bats, two adult humans, one male and one female, were also present in the room during the experiments. The 3D spatial position of the bats and humans was recorded using a modified version of a commercial RTLS, similar to that used previously^20^. The system was composed of mobile tags (DWTAG100) that were identified and localized at a 100-Hz sampling rate by 16 static anchors (DWETH101) placed on the walls and ceiling of the room, providing reference locations for the system. Anchors and tags communicated through ultra-wideband pulses. An additional anchor (custom DWETH101) was used to record an external synchronization signal (see below). Each lightweight (~2.9 g) transceiver tag was powered with a lithium polymer battery (~15 g total). For bats, the transceiver tag was directly mounted on the neural implant. For the humans, one tag was placed in the right laboratory coat pocket and one each on the left and right wrists, for a total of three tags per human (Extended Data Fig. 1). The system communicated with a computer outside the experiment room through the User Datagram Protocol and was configured and operated through a web-based user interface running on Ubuntu 18.04 Bionic. Data were recorded and saved with custom scripts in Python. The spatial resolution of the system was measured on a subset of the experimental sessions in which one bat was simultaneously tracked with two recording systems: the RTLS and a precise marker-based motion capture system (Cortex v6.2.13.1751 (Motion Analysis) run in MATLAB 2021a (MathWorks))^20,30,31^. The spatial resolution on tracked bats and humans was in the range 10–20 cm. Periodic clock pulses generated by a Master-9 device (A.M.P.I.) were used to create a timing signature that served as a common frame of reference for all of the recording systems (tracking and neural recordings). Four tripods were positioned around the room to demarcate the possible locations of the human experimenters. The tripods were all set to the same height and did not change positions during the entire experiment. Video of the room at the tripod and resting locations was acquired using infrared cameras at 25 fps (Basler ace acA800-510um; Basler Pylon image acquisition software (v6.2.0.8205)). Both humans wore the same standard personal protective equipment, which included a white laboratory coat, a blue hair bonnet, a blue surgical mask, blue gloves, blue shoe covers, clear protective glasses and a protective yellow leather glove on the left hand. When standing at a tripod location, each human placed their left palm up on the tripod and used their right hand to dispense 0.3 ml of banana smoothie using a wireless manually activated motorized feeder, when appropriate (Arduino Mega Rev3; Adafruit Motorshield 1438; Arduino IDE v1.8.19). Experimenter hand sizes and gloves were highly similar, which served as structurally and visually indistinguishable landing platforms for the bat. All bats were mildly food-restricted (>90% of their baseline weight) before the task sessions. Before the start of the neural recording, the bats were introduced to the experimental setup, trained on the behavioral task described below and were familiar with both experimenters.

### Behavioral task

Two experiments were performed in each recording session. In experiment 1 (~90 min duration), bats were trained to freely fly to either human for a reward. In experiment 2 (~30 min duration), bats hung in their preferred rest locations while the humans approached the bats to feed or handle them. Experiment 1 consisted of several 15-min blocks. During each block, the experimenters picked a pair of tripod locations to stand at for 5 min, then swapped locations for the next 5 min and returned to their original locations for the final 5 min of the block. The blocks were counterbalanced such that each human spent an equal amount of time at each tripod location over the course of the session. To control for potential glove-specific cues from the yellow ‘landing platform’ gloves, the experimenters swapped yellow gloves every block. Each time the humans changed locations, they walked radially inward from their locations to the center of the room, and radially outward to their new locations. If a bat happened to fly while the humans were walking, the humans paused their movement. The bats could freely fly to any tripod in the room, but they were only rewarded if they landed on the experimenter’s hand on the tripod. When a bat landed on an experimenter’s hand, a single reward was dispensed, and the next feed could only be triggered by the bat flying away and returning. If the second bat happened to land on the same experimenter before the first bat left, the reward was dispensed only to the second bat. For two of the four bats, two additional tripod locations were available (six locations total) for the humans to stand at, and human 1 gave 0.3 ml of reward, whereas human 2 gave 0.1 ml of reward. This difference in reward did not significantly impact the ratio of visits to each human (Extended Data Fig. 2b; bat 3 and bat 4). The ratio of the number of visits to human 1 versus human 2 was calculated for all sessions for all bats within an experimental paradigm (*n* = 20 sessions for the four-tripod paradigm and *n* = 11 sessions for the six-tripod paradigm (two-tailed *t*-test, *P* = 0.1). Experiment 2 immediately followed experiment 1. During experiment 2, the humans stood together at the center of the room and randomly took turns performing traverses along a fixed, arced path to either feed or handle the bats hanging at their preferred rest location. Handling consisted of a standard mild restraining grasp while the bats were hanging, which lasted the same amount of time as the reward delivery. All traverses were performed when both bats were hanging in the preferred rest location, and each human rewarded the same bat across all sessions (their ‘designated bat’). During each traverse, one experimenter walked from the center of the room in an arced path towards the bats, paused below the bats, fed their designated bat and handled the other bat, and walked in an arced path back to the center of the room. A slight variant of this experimental paradigm was performed for two of the four bats, in which the humans waited at tripods instead of the center of the room but still performed the same traverse from the center of the room to the bats and back to the center of the room. In this variant, the humans did not have a designated bat to handle or feed, and instead randomly chose which bat to reward during each trial (*n* = 20 sessions for the designated-bat paradigm, and *n* = 11 sessions for the random paradigm).

### Microdrive implant procedure

Surgical procedures for electrophysiology implants were performed similarly to those described previously for Egyptian fruit bats^20,31–33^. A lightweight four-tetrode microdrive (Harlan 4 drive; Neuralynx) was implanted over the right hemisphere of each bat. Tetrodes were made from four strands of platinum–iridium wire (17.8 μm diameter, HML insulated) and assembled as described previously. Each tetrode was loaded into a telescoping polyamide tube assembly inside the microdrive, and each tetrode was independently manipulable (~5 mm travel). Approximately 12–16 h before surgery, the tips of the tetrodes were cut to the same length and plated with Gold Plating Solution (Neuralynx), bringing the impedance of individual wires down to 0.3–0.6 MΩ. For the surgical procedure, anesthesia was induced with an injectable cocktail of ketamine, dexmedetomidine (Atipamezole) and midazolam (Flumazenil). The bat was placed in a stereotaxic apparatus (Model 942; Kopf) where a continuous supply of oxygen was provided, and anesthesia was maintained by injection (~once per hour) of a cocktail of dexmedetomidine, midazolam and fentanyl. The depth of anesthesia was continuously monitored by reaction to a toe pinch test and by measuring the bat’s breathing rate. Body temperature was measured with a rectal temperature probe and kept at approximately 35 °C with a regulated heating pad. After the proper anesthetic depth was reached, the skull was exposed, and the surrounding skin and tissue were retracted. The skull was then cleaned of any connective tissue and scored to improve adhesion and mechanical stability. A bone screw (19010-00; FST), with a short piece of stainless-steel wire (0.008 in. coated; A-M Systems) soldered to the screw head, was inserted into the frontal plate of the skull and served as ground for the microdrive. Four shorter bone screws (M1.59, 2 mm, stainless steel) were placed to further stabilize the implant. A circular craniotomy of 1.8 mm was made in the skull above the right hemisphere hippocampus CA1 at 5.4 mm anterior to the transverse sinus that runs between the posterior part of the cortex and the cerebellum and 3.7 mm lateral to the midline. The craniotomy was covered with a biocompatible elastomer (Kwik-Sil; World Precision Instruments) to protect the brain while the skull and the base of the screws were covered with a thin layer of bone cement (C&B Metabond; Parkell). The Kwik-Sil was then removed to perform the durotomy and lower the microdrive, with tetrodes fully retracted, into the craniotomy. The microdrive was lowered to the surface of the brain to create a tight seal, and the remaining exposed part of the brain was covered with Kwik-Sil. Dental acrylic was applied in layers to secure the microdrive to the screws and the skull. A ground wire from the microdrive was connected to the wire from the ground screw, and the whole connection was embedded in the dental acrylic. Once the acrylic was dry, all four tetrodes were lowered to their initial positions, approximately 800 μm below the cortical surface. To conclude the surgery, reversal agents were given to counteract the dexmedetomidine and midazolam. After the bat woke fully from the anesthesia, meloxicam (Metacam; Boehringer Ingelheim), an oral analgesic, was administered. After surgery, analgesics (3 days) and antibiotics (7 days) were given daily until complete recovery.

### Electrophysiology data acquisition, preprocessing and spike sorting

After microdrive implantation, tetrodes were lowered in small increments every day over a period of 1–2 weeks, advancing towards the pyramidal layer of the dorsal hippocampus (CA1). The pyramidal cell layer was initially determined by the detection of high-frequency ‘ripples’ in the local field potential signal, together with a transient (50–100 ms) increase in multi-unit activity. All tetrode adjustments were made while the bat was swaddled in a small fabric bag. Neural activity from the tetrodes was checked every day by connecting the bats’ microdrive to a wired recording system (Digital Lynx; Neuralynx) before and after experimental sessions. At the end of each session, one or more tetrodes were moved (20–160 μm) to sample a different group of neurons (upon tissue stabilization the following day). Tetrode movements were timed to ensure maximal time for stabilization of the tissue before the next day’s recordings. Tetrode positions were verified posthumously with histology (see below). To record neural activity while the bats were freely flying, we used a wireless neural data-logging system (‘neural logger’; MouseLog16, vertical version, Deuteron Technologies) similar to that used previously^20,31^. The logger was housed in a custom 3D-printed case, along with the RTLS tag and two lithium polymer batteries (one for the logger and one for the RTLS tag; minimal duration, 150 min), and connected to the electrical interface board of the microdrive at the beginning of each recording. Implanted bats used in the experiment weighed more than 110 g and could fly normally while equipped with the neural loggers and RTLS tags, as expected based on previous experiments using wireless recording systems^20^. Electrical signals from the four tetrodes (16 channels) were amplified (200×), bandpass filtered (1–7,000 Hz), sampled continuously at a frequency of 31.25 kHz and stored on an SD memory card on the logger, with a voltage resolution of 3.3 μV. Wireless communication between the neural logger and a static transceiver ensured proper synchronization and allowed basic monitoring and configuration using software (Deuteron Technologies). At the end of the recording session, data from the loggers were extracted and saved. Spike sorting was performed as described previously^20,21,31^. In brief, recorded voltage traces were filtered (600–6,000 Hz), and putative spikes were detected by thresholding (3 s.d.) the filtered trace. Putative spike waveforms (32 samples, peak at the eighth sample) were fed into the cluster sorting software (SpikeSort 3D; Neuralynx). Manual sorting was performed using spike amplitude and energy as the main features. We quantified interneurons as units having a mean firing rate of >5 Hz, and identified a total of 24 interneurons. We found similar results between principal cells and interneurons. Specifically, 70.8% (17 out of 24 putative interneurons) carried significant 2D spatial information during flight, 45.8% (11 out of 24 putative interneurons) modulated activity depending on the identity of the human landing target and 29% (7 out of 24 putative interneurons) carried significant spatial information for the position of a human traversing the environment. Thus, the fractions of interneurons that (1) carry significant 2D spatial information, (2) are human modulated and (3) carry information about the position and identity of the human are representative of the fractions in the general unit population and similar to those found in the pyramidal unit population.

### Histology

At the end of the electrophysiology experiments, the bats were given a lethal dose of sodium pentobarbital and perfused transcardially with 200 ml PBS (0.025 M, pH 7.4), followed by 200 ml of fixative (3.7% formaldehyde in PBS). The microdrive and tetrodes were left in place throughout the perfusion process. Half an hour after the perfusion was complete, the tetrodes were fully retracted, the microdrive was removed and the brain was carefully dissected and stored in the fixative solution for 1–2 days. Following fixation, the brain was transferred to a 30% sucrose solution in PBS for 1–3 days. The brain was sliced into 40-μm coronal sections on a freezing stage using a microtome (Microm HM 450, Thermo Fisher Scientific). Following previously described procedures^20^, the sections were then stained using DAPI and antibodies against PCP4 and IBA1. In brief, sections were permeabilized in PBS plus 0.3% Triton-X (PBS-X) and incubated in a blocking solution (PBS-X plus 10% donkey serum) for 2 h. The sections were then incubated overnight at 4 °C with primary antibodies (goat anti-Iba1, 1:500 dilution, ab5076, Abcam; rabbit anti-PCP4, 1:500 dilution, HPA005792, Sigma). After primary incubation at 4 ºC, the sections were washed in PBS-X and incubated for 120 min at room temperature with secondary antibodies (donkey anti-goat Alexa-647, 1:1,000 dilution, A32849, Invitrogen; donkey anti-rabbit Alexa-488, 1:1,000 dilution, A-21206, Invitrogen). DAPI (1:10,000 dilution, Thermo Fisher Scientific) was added during the last 10 min of secondary incubation.

Sections were washed in PBS-X and cover-slipped using an aqueous mounting medium (ProLong Gold Antifade Mountant, Thermo Fisher Scientific). Fluorescent images of each section around the implant coordinates were acquired using an Axioscan Slide Scanner (Zeiss). The location of the tetrodes used for the analyses were visualized and localized to the dorsal hippocampal area CA1.

### Data analysis

All analyses were conducted using custom code in MATLAB (2022a).

#### Processing of positional data during tasks

##### Preprocessing of bat tracking data and basic analysis of positional features

The positions of all bats recorded by the RTLS were smoothed using local quadratic regression (1-s window). For three out of the four bats, a velocity threshold of 0.5 m s^−1^ was used to segment a bat’s session into rest and flight epochs. The fourth bat was tracked exclusively with the marker-based motion capture system (Cortex, Motion Analysis), and the same 0.5 m s^−1^ threshold was used to segment the bat’s session into rest and flight epochs. To ensure precise capture of flight initiation and landing, flight epochs were manually inspected and trimmed based on the convergence of velocity in the *x*, *y* and *z* directions to zero. Bats tended to rest in a handful of locations, almost exclusively in two upper corners of the room (Extended Data Fig. 9). While in their rest location, bats did not typically crawl to different places on the wall. Epochs when the bats were confirmed to be stationary in a preferred rest location were isolated using a velocity threshold of <0.4 m s^−1^ and when positional data indicated that the bat was <200 cm from the centroid of a preferred rest location for that session.

##### Preprocessing of human tracking data and basic analysis of positional features

Positions of humans were tracked with three RTLS tracking tags, one positioned on the right hand (reward delivery hand), one positioned on the left hand (landing pedestal hand) and one in the right laboratory coat pocket. A moving median filter (2 s) was applied to the tracking data obtained from the tag in the coat pocket. The beginnings of human traverse trials were defined as the times at which the velocity of the human’s laboratory coat tag exceeded the defined threshold (0.4 m s^−1^), and the human was <0.1 m from the *xy* coordinates of the traverse start position. The end of traverse trials were defined as >12 s after the start of a traverse trial, when the velocity of the human’s laboratory coat tag dipped below threshold (0.4 m s^−1^) and its distance was <0.3 m from the *xy* coordinates of the traverse start position. Data from rare cases in which humans paused mid-traverse and the velocity dipped below 0.2 m s^−1^ were discarded. Human velocity was calculated using positional data from the tag in the coat pocket, as it was unaffected by hand and arm acceleration and deceleration (Supplementary Fig. 6). All periods when the human was handling or administering the reward were excluded from the analysis and were identified as follows: the peaks of accelerometer movement from the tags on the right and left hands were used to identify putative reward initiations (when the humans raised their hands up to deliver the reward from a syringe). Reward delivery and bat handling were further verified by manual inspection of video. Timestamps that marked the start and end of the handling and reward epochs were buffered with an additional 100 positional samples to ensure no artifactual signal was included in the analysis of human traverses.

#### Place fields and spatial information

##### Spatial information in 2D during self-motion

For the analysis of spatial firing fields across all flights, we considered only active cells (*n* = 259 from four bats), with a minimum firing rate of 0.2 Hz, a minimum of 12 flights and a minimum of five flights with at least five spikes. We focused on the spatial firing in the *xy* plane (parallel to the ground), where most of the positional variance was concentrated. To compute 2D spatial firing-rate maps, we projected all positions during flight onto the *xy* plane and calculated occupancy-normalized firing rates. To do this, we binned the 2D area of the room into spatial bins of a fixed size (0.15 × 0.15 m^2^), calculated the time spent in each bin (occupancy) and counted the number of spikes (spike-count) in each bin. We smoothed both the spike count map and occupancy map with a Gaussian kernel (*σ* = 1.5 bins) and calculated their ratio, bin by bin, thus obtaining the firing rate per bin. Spatial bins in which the bat spent <150 ms were invalidated (white pixels in rate maps; for example, Extended Data Fig. 3c), unless surrounded by at least one valid bin. Spatial information per spike^34^ was calculated by summing across all valid bins:

$$\mathrm{SI}=\sum _{i}\frac{{p}_{i}{\lambda }_{i}}{\lambda }{\log }_{2}\frac{{\lambda }_{i}}{\lambda }$$

where *p*_*i*_ is the probability of being in bin *i*, *λ*_*i*_ is the firing rate in the same bin and *λ* is the average firing rate across all bins. Cells were classified as significantly spatially informative using a shuffling procedure. We compared the empirical value of the spatial information to a spike-shuffled distribution, which was generated by randomly shifting the timestamps of the cell’s spike train circularly relative to behavior (after removing rest epochs) and used to calculate shuffled spatial information. The shuffle procedure was repeated 1,000 times for each neuron. Significant place cells were defined as active neurons for which the empirical value of the spatial information exceeded the upper 95% confidence interval of its shuffled distribution.

##### Spatial information in 1D during self-motion (trajectories)

Many flights that the bats execute are idiosyncratic, repeated paths that emerge as the animal explores the room (Fig. 1b). We took advantage of this behavior and calculated spatial firing maps along tightly confined repeated trajectories. Flights were clustered into trajectories by using an analogous approach to that described previously^19,20,31^. In brief, all trajectories were spatially downsampled to seven points per flight (first and last points corresponded to the take-off and landing positions, respectively). The Euclidean distance between downsampled flights was used as a measure of flight similarity, and similar flights were clustered together using agglomerative hierarchical clustering. The linkage distance was set between 1.2 m and 1.4 m for each session, after manual inspection of trajectories. One-dimensional spatial firing fields were calculated for each trajectory and neuron with at least seven flights, a minimum of four flights with spikes and a minimum of 15 spikes across all flights (*n* = 247 cells from four bats). To compute the 1D fields, we used a procedure similar to the one for 2D maps, except applied in only one dimension. To do this, flights of each analyzable trajectory were rescaled and binned between take-off and landing such that each bin’s edges were defined by the distance from take-off along the flight trajectory (bin size, 0.15 m). The amount of time spent in each bin was calculated (1D occupancy map), and the number of spikes in each bin were counted (1D spike map). The 1D occupancy maps and 1D spike maps were smoothed with a Gaussian window (seven samples), and spatial information was calculated across 1D bins as described above. Similar to the process described for the 2D rate maps, a shuffling procedure was used to assess the significance of the spatial information of each 1D field. To construct the shuffle distribution of spatial information values, each flight in the trajectory was rescaled (as above), the spike train was randomly circularly shifted relative to the rescaled position and the final shuffled spike map was obtained by counting the number of spikes in each bin across all circularly shifted flights (as above). This shuffled spike map was smoothed with a Gaussian window (seven samples), and the bin-by-bin ratio of the shuffled spike map and 1D occupancy map produced the shuffled rate map. This shuffle was repeated 1,000 times, and the resulting spatial information values built the distribution to which the empirical spatial information value was compared. Significant 1D fields were defined as those for which the empirical value of the spatial information exceeded the upper 95% confidence interval of its shuffled distribution, after Bonferroni correction for the number of trajectories examined for that neuron. The stability of 1D fields within a session (Extended Data Fig. 3) was measured by splitting each path into even and odd flights (trials), separately calculating 1D fields on each half (Extended Data Fig. 3a) and calculating the Spearman correlation between corresponding halves (Extended Data Fig. 3b). The stability of 1D fields within a session across human landing targets was measured by splitting each path into flights to experimenter 1 and flights to experimenter 2, separately calculating 1D fields on each set of flights (Supplementary Fig. 2a) and calculating the Spearman correlation between corresponding sets (Supplementary Fig. 2b).

#### Correlation of flights within and across human landing targets

To quantify the similarity of flights of a given trajectory within and across human landing targets, we calculated the Pearson correlation between pairs of flights from trajectories by concatenating the *x*, *y* and *z* coordinates (Fig. 1c). For every trajectory that had at least four repeated flights to each human, we calculated the intra-human and inter-human trajectory correlation as follows. To obtain the intra-human trajectory correlations, we calculated the Pearson correlation of each flight of a given trajectory and human landing target to each other flight of that trajectory and the same human landing target. Correlations from all the human landing target and trajectory combinations were then pooled together. To obtain the inter-human trajectory correlations, we calculated the Pearson correlation of each flight of a given trajectory and human landing target to each other flight of that trajectory to the other human landing target. Correlations from all trajectories were then pooled together.

#### Correlation of traverses between and across humans

To quantify the similarity of human traverses, we calculated the Pearson correlation between the concatenated *x* and *y* coordinates of pairs of traverses (Fig. 2c). To obtain a measure of similarity between traverses executed by one human, we calculated the Pearson correlation of all traverses that one human performed to all other traverses that same human performed. To quantify the similarity of traverses across humans, we calculated the Pearson correlation of each traverse that one human performed to each other traverse that the other human performed. We quantified the similarity of the humans’ velocity during traverses by calculating the Pearson correlation between the two humans’ velocity profiles (concatenated *x*, *y* and *z* velocity) (Supplementary Fig. 6).

#### Effect of experimenter identity on firing modulation of hippocampal neurons in flying bats

To quantify whether a neuron was significantly modulated by the identity of the human at the landing and/or take-off locations, we calculated the difference in mean firing rate of flights that ended and/or started in the same location, but with different humans standing at that location. Inspection of the peak-normalized sorted plot of 1D place fields suggested that peak activity clustered around take-off and landing (Extended Data Fig. 3a), so we chose a 2-s window at take-off (−1.75 to +0.25 s) or at landing (−0.25 to +1.75 s) to calculate the mean difference in firing rate. To determine whether a cell was significantly modulated by the identity of the human landing target at a given landing location, we performed a permutation test as follows (this process was then done for all take-off locations). For each landing location that had at least four trials to each human, 15 spikes across all trials in the landing time window and at least four flights with spikes in the landing time window, we calculated the difference in mean firing rates obtained in the landing time window to each human. We then constructed a shuffled distribution of mean firing rate differences by shuffling the label of which human was the landing target for each trial and taking the difference in mean firing rates of two subsets of the shuffled-label data with equal sizes to the empirical data. The shuffling was performed 1,000 times, or for as many permutations as the number of trials per human permitted (maximum permissible permutation test resolution for inclusion was *P* = 0.02). Neurons were significantly modulated by the human landing target if the empirical value of the difference in mean firing rates exceeded the upper 95% confidence interval of the shuffled distribution, after Bonferroni correction for the number of landing and/or take-off locations examined. For the analysis that explicitly excluded any flights in which the other bat in the room was present at landing, we performed the same procedure as above but only for landing locations where the inclusion criterion was met after excluding all trials when the other bat was at the landing location. The other bat was classified as present at a landing location if the Euclidean distance of the other bat’s position to the coordinates of the tripod at that location was <200 cm and the bat’s velocity dipped below the flight detection threshold (0.4 m s^−1^).

#### Effect of the presence of the other conspecific at landing on firing rate during flight

To quantify whether a neuron was significantly modulated by the presence of the other conspecific at the landing location, we used the same permutation test performed above for the human landing target. We calculated the difference in mean firing rate in the window at landing (−0.25 to +1.75 s aligned to landing) between flights to the same locations where the other conspecific was either present or absent. We compared this empirical value to a shuffled distribution obtained using the permutation test, shuffling the labels of whether the conspecific was absent or present at landing. Neurons were significantly modulated by the presence of the conspecific at landing if the empirical value of the difference in mean firing rates exceeded the upper 95% confidence interval of the shuffled distribution, after Bonferroni correction for the number of landing locations examined for that neuron. We classified the other bat as present at a landing location in the same manner as described above.

#### Spatial information for the position of the other bat in the room during recorded bat rest

To assess whether neurons carried significant spatial information about the position of the other bat in the room, we calculated the spatial information of 1D rate maps using the neural data of the stationary recorded bat and the 1D occupancy maps of trajectories (linearized and binned, described above) executed by the conspecific. We clustered the flights of the conspecific into trajectories using the agglomerative hierarchical clustering method (see above). We then excluded any flights from analyses in which the recorded bat was not in a preferred resting location. The recorded bat was confirmed to be in a preferred resting location if velocity was below a threshold of 0.4 m s^−1^ and the Euclidean distance of the bat to the centroid of the preferred resting location was <200 cm. Preferred resting locations were determined by performing *k*-means clustering on all positional data for which the velocity threshold dipped below 0.4 m s^−1^ and taking the centroids of the resulting clusters. Spatial firing was calculated for each conspecific trajectory and recorded bat neuron for which there were at least seven flights, a minimum of four flights with spikes and a minimum of 15 spikes across all flights (*n* = 130 cells from four bats). We computed the 1D rate maps in the same manner as the self-motion 1D trajectories above and applied Bonferroni correction for the number of trajectories examined for that neuron. The significance of the spatial information was assessed by comparing the empirical value to a shuffled distribution. The shuffled distribution was constructed in the same manner as described above for the shuffled distribution of the 1D rate maps.

#### Spatial information for human position during human movement and recorded bat rest

To assess whether neurons carried significant spatial information about the position of the humans in the room, we performed an analysis similar to the one for assessing 2D spatial information described above. First, we asked whether neurons from the stationary recorded bat carried significant spatial information for the position of any human during the stereotyped traverse to and away from the bats hanging in a preferred rest location. During experiment 2, only one human was ever moving at a time, and we included all times when a human was moving but was not handling or administering the reward (see above for how human traverses were defined). We also excluded any times when the recorded bat was not in the preferred resting location (see above for how a bat was determined to be at a preferred resting location). Rate maps were calculated from the human 2D occupancy map and recorded bat spike data. Only sessions with at least six human traverses, at least four traverses with spikes and at least 15 spikes over all traverses were included in the analysis. We projected all human positional data onto the *xy* plane, binned the positional samples into 0.15 × 0.15 m^2^ bins and calculated the amount of time spent in each bin (occupancy map). We then counted the number of spikes from the recorded bat that occurred in each bin to obtain the spike map. We smoothed both the spike map and occupancy map with a Gaussian kernel (*σ* = 1.5 bins) and calculated their ratio bin by bin, thus obtaining the firing rate per bin. Spatial bins for which the human spent <1 s were invalidated (white bin in rate maps), unless surrounded by at least one valid bin. Spatial information was calculated from the 2D firing rate map and compared to a shuffled distribution. The shuffled distribution was obtained by circularly shifting the spike train of the recorded bat relative to human movement (with periods between traverses removed), counting the number of spikes in each spatial bin (shuffled spike map), computing the bin-by-bin ratio of the shuffled spike map to the 2D occupancy map (shuffled rate map) and calculating the spatial information from that shuffled 2D rate map. Neurons carried significant spatial information for human movement if the empirical value of the spatial information exceeded the upper 95% confidence interval of its shuffled distribution. To assess the spatial information for each human’s movement independently, we performed the same analysis as above but with occupancy maps for traverses of either experimenter 1 or experimenter 2 and applied Bonferroni correction for the number of experimenters examined per neuron (Fig. 2e). Only traverses with at least six trials for a given human, at least four trials with spikes and 15 spikes total across all traverses were included in the analysis. For all neurons that carried significant spatial information for just one human, we compared the normalized spatial information for the human for which the spatial information was significant (preferred human) to the normalized spatial information of the other human (non-preferred human) (Wilcoxon signed-rank test, *n* = 43 neurons, *P* = 1.1 × 10^−8^; Fig. 2f). Normalized spatial information is the empirical spatial information divided by the mean of the spatial information values calculated from spike-shuffled trials^35^. Then, for each neuron, we compared the normalized spatial information value to the normalized spatial information value calculated from the rate map that excluded all epochs when the other conspecific was flying (Wilcoxon signed-rank test, *n* = 43 neurons, *P* = 0.44; Extended Data Fig. 10b). Flight epochs were determined using the method described above for identifying periods of flight in the bats.

#### Conjunctive code for space and experimenter identity during self-motion

To quantify the extent to which neurons significantly modulated their activity during flight depending on the human landing target at multiple different locations in the room, we first identified which neurons had at least four locations with enough flights to and/or from each human to be analyzed. We then counted the number of locations for which each neuron significantly modulated its activity depending on the identity of the human (Fig. 1g). Then, to quantify how factors of human identity and location contributed to the firing rate of a neuron at landing, we used a simple linear model to predict the mean firing rate around landing by using three predictors: a non-ordinal categorical variable encoding the identity of the human at landing, a non-ordinal categorical variable encoding the landing location, and their interaction term (‘lmfit’ in MATLAB; Extended Data Fig. 6a). The categorical variable for landing location is the assigned number of the tripod at which the bat landed. This was determined by calculating the Euclidean distance of the bat’s position at the end of each rewarded flight to every possible tripod landing location (1–4) and taking the tripod with the minimum distance. A model comparison was performed to identify, for each neuron, which variables significantly improved the prediction of firing rate upon landing (‘anova’ in MATLAB; significance threshold, *P* < 0.05). This model allowed us to disambiguate a purely additive coding for human and location from a conjunctive coding of human and location. In total, 134 neurons were modeled, and seven neurons were not included in further analyses because there were no significant variables that improved the prediction of firing rate at landing (*n* = 127 neurons). Each neuron was classified as ‘additive’, ‘conjunctive’, ‘human only’ or ‘location only’. ‘Additive’ neurons had significant human and location variables, but not significant interaction terms. ‘Conjunctive’ neurons had significant human and/or location terms, as well as a significant interaction term. ‘Human only’ neurons had a significant human term, but not significant location or interaction terms. ‘Location only’ neurons had a significant location term, but not significant human or interaction terms. We then compared these results to those obtained using a permutation test classifying a neuron as modulated by the human identity upon take-off and/or landing (see above; Extended Data Fig. 6b).

#### Effect of the reward quantity on unit modulation at landing

To examine whether there was a global effect of reward quantity on neural responses around landing on different human targets, we calculated the peak firing rate change around landing, the mean firing rate change immediately upon landing (0 to +1.75 s landing at 0) and the Spearman correlation between average firing rates to different human landing targets for a given unit and landing location (Supplementary Fig. 5). Peak firing rate change was calculated by dividing the peak firing rate in the same window as used previously (−0.25 to +1.75 s around landing) by that unit’s baseline firing rate. Mean firing rate change was calculated by dividing the mean firing rate in the window immediately after landing by that unit’s baseline firing rate. We used the same inclusion criteria as in previous analyses, including only units × landing locations × humans with at least four flights with spikes and at least 15 spikes across all flights.

#### Remapping analysis

##### Remapping on specific trajectories

We calculated the correlation, distance between peaks and remapping scores for the 1D linearized rate maps of a given trajectory to different human landing targets (Extended Data Fig. 5). For a pair (trajectory × unit) to be included in the analysis, it had to meet the same criteria that was used to examine the 1D spatial information above (at least four flights with spikes and at least 15 spikes across all flights), and had to carry significant 1D spatial information. Linearized rate maps were computed in the same manner as described for calculating the 1D spatial information above. The correlation was the Pearson correlation between 1D rate maps to different human landing targets. The distance between peaks was the distance between maximum values of the linearized rate maps. The remapping score was calculated as follows^36^: for a given unit and trajectory, we obtained the mean firing rates during the 1D linearized rate maps to different human landing targets; the score was defined as the unsigned difference between those rates, divided by their sum. A score of 0 indicates no rate change, whereas a score of 1 indicates that one rate value dominates the other. The empirical distributions of the correlation, distance between peaks and remapping scores were compared to their respective null distributions. The null correlations were calculated by taking the Pearson correlation between 1D linearized rate maps of non-paired units (one per unit) for a given trajectory to different human landing targets. The null distance between peaks was calculated by taking the distance between maximum values of the linearized rate maps of non-paired units for a given trajectory to different human landing targets. The null remapping score was calculated by shuffling the human landing target labels between flights of a given trajectory and calculating the rate maps for paired units across the trial-shuffled rate maps. It is important to note that the remapping score null distribution represents no rate remapping, as the trials are randomly chosen across human landing targets. By contrast, the null distributions for correlation and distance between peaks represent global remapping, as they compare the rate maps of non-paired units (random movement of place fields). If the spatial profile of the responses to different experimenters is much more similar (high correlation, low distance between peaks) than those predicted by global remapping, then this suggests an absence of global remapping. If the difference in firing rates (remapping score) is significantly higher than predicted by the null hypothesis, then this suggests a phenomenon resembling rate remapping.

##### Remapping on 2D rate maps between contexts

Classical remapping analyses compare the rate maps across contexts in an experiment. In the present study, humans stood at alternating locations in the room for extended periods of time, creating ‘contexts’ where the humans were standing in a given configuration (context 1 is experimenter 1 at location A and experimenter 2 at location B, and context 2 is experimenter 1 at location B and experimenter 2 at location A; Extended Data Fig. 5). To compute remapping metrics across these contexts, we defined a context as all of the occupancy and spikes that occurred when the humans were standing in a given configuration in the room. If there were multiple contexts in which sufficient flights and spikes occurred, the top two contexts with the most occupancy were used. For a pair (unit × contexts) to be included, the fraction of pixels shared between the two contexts’ rate maps had to be at least 0.3, there had to be at least 20 spikes in total within a context and the unit had to carry significant spatial information. The 2D rate maps for each context were computed as described above in the section detailing the 2D rate map calculation. The correlation, distance between rate map centroids and remapping scores were then computed between rate maps. The remapping score was calculated in a similar manner as the 1D case: the unsigned difference in mean firing rates of the 2D rate maps was divided by their sum. The center of mass movement was the Euclidean distance between the center of mass of each rate map, as identified by the MATLAB ‘regionprops’ function. The correlation was simply the Pearson correlation between rate maps. The null distributions for correlation and center of mass movement were constructed in a similar manner as the 1D case: the correlation between, or the distance between centers of mass of, two rate maps of non-paired units (one per unit) from each context was used. The null distribution for the remapping score was also constructed in a similar manner as the 1D case: a random subset of flights from each context was used to construct the rate maps, and the remapping score was computed across trial-shuffled maps for paired units. As explained for the remapping analysis of specific trajectories, the null distribution of the remapping score represents no rate remapping, whereas the null distributions of the correlation and distance between peaks represent the presence of global remapping.

##### Temporal stability of human-modulated units

To determine whether the modulation associated with the human landing target was present from the first trial or emerged as the session progressed, we calculated the correlation between mean firing rates of the first and second halves of flights along each 1D linearized trajectory to a given human (Supplementary Fig. 3). This distribution of correlations was compared to a null distribution constructed from the correlation between randomly chosen subsets of flights from that trajectory and human. The null represents what would be expected if the modulation were present from the first trial and did not evolve over time.

#### Statistical analysis

No formal methods were applied to predetermine sample sizes, and adopted sample sizes were similar to those used in similar studies. No randomization of experimental sessions was performed, and no blinding to experimental conditions was implemented during the analysis. All statistical comparisons were performed using nonparametric tests (permutation test, Wilcoxon signed-rank test, Kolmogorov–Smirnov test, or bootstrap test) unless otherwise stated. The tests were two-tailed. Where appropriate, adjustments for multiple comparisons were performed using Bonferroni correction.

### Reporting summary

Further information on research design is available in the Nature Portfolio Reporting Summary linked to this article.

## Online content

Any methods, additional references, Nature Portfolio reporting summaries, source data, extended data, supplementary information, acknowledgements, peer review information; details of author contributions and competing interests; and statements of data and code availability are available at 10.1038/s41593-024-01690-8.

## Supplementary information

Supplementary Information Supplementary Figs. 1–6 and legends.

Reporting Summary

## Source data

Source Data Fig. 1 Source data for Fig. 1c,e,g.

Source Data Fig. 2 Source data for Fig. 2c,f,g.

## Data Availability

The data presented in this study will be made available from the corresponding author upon request. Source data are provided with this paper.

## Code availability

All analyses were conducted using custom code in MATLAB 2022a. The code generated in the current study is available from the corresponding author on request.
