# Supplementary material for: Neural representation of human experimenters in the bat hippocampus
Source: Nat Neurosci. 2024 Jul 2;27(9):1675–9. doi: 10.1038/s41593-024-01690-8 (PMC11374686; doi:10.1038/s41593-024-01690-8)
Supplement: Supplementary file 1 — Supplementary Figs. 1–6 and legends. [file 41593_2024_1690_MOESM1_ESM.pdf]

nature neuroscience

Supplementary information https://doi.org/10.1038/s41593-024-01690-8

Neural representation of human experimenters in the bat hippocampus

In the format provided by the authors and unedited

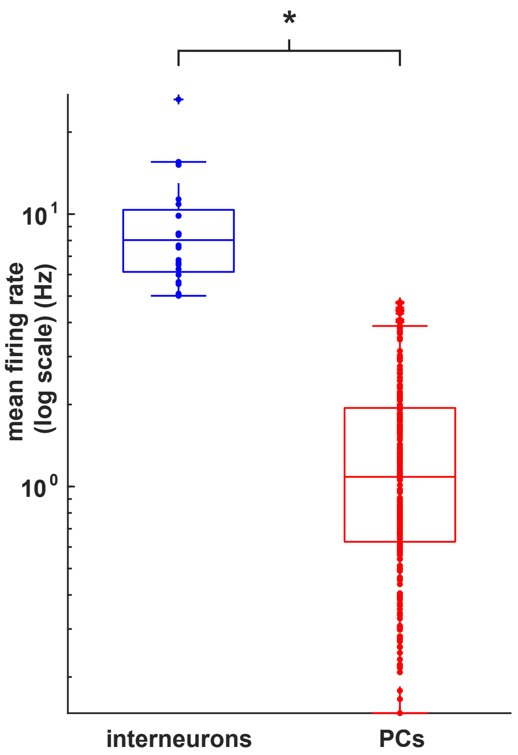

Supplementary Fig. 1 | Distribution of mean firing rates for putative interneurons v.s. principal units. A total of 24 putative interneurons were identified based on a threshold of > 5Hz mean firing rate over the course of the session. Blue boxplot indicates median firing rates (log scale) of interneurons (n = 24). Red boxplot indicates median firing rates (log scale) of putative principal cells (n = 251). Box bounds indicate 25th and 75th percentiles, error bars indicate minima and maxima, outliers indicated with a cross. Single asterisk indicates significant difference in firing rates between putative interneurons and principal cells by two-sided Wilcoxon rank sum test ($p = 1.7 \times 10^{-16}$).

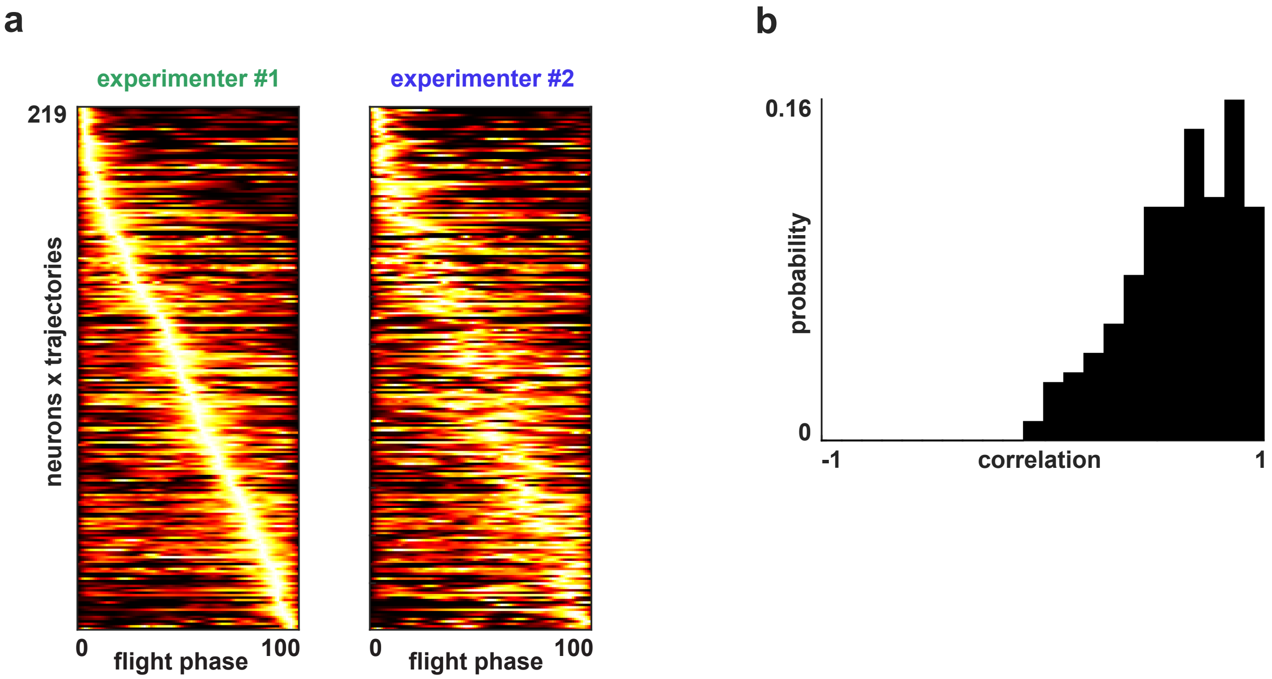

Supplementary Fig. 2 | Stability of place fields across human landing targets.
a. Left: Peak-normalized 1D rate maps for all spatially significant neurons x trajectories (trials to human landing target #1) sorted by peak activity over time (n = 219 neurons x trajectories). Right: 1D rate maps of all spatially significant neurons x trajectories (trials to human landing target #2), sorted according to the order of trials to human landing target #1. **b.** Distribution of Spearman correlations between pairs of 1D rate maps constructed from trials to human landing target #1 and #2, for each unit x trajectory (n = 219 neuron x trajectory pairs).

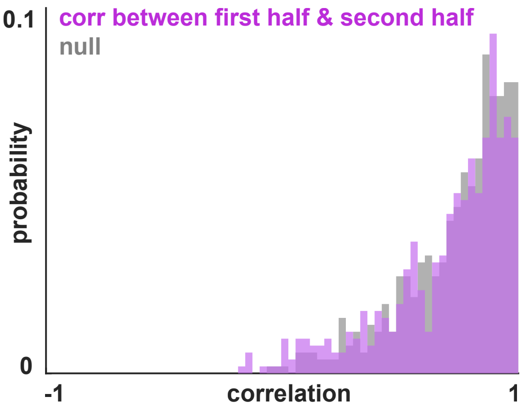

Supplementary Fig. 3 | Distribution of correlations between first and second halves of trajectories to each human.

Distribution of correlations between 1D linearized mean firing rates for first and second halves of a given trajectory to a given human (purple) versus the null (grey) (n = 527 analyzable neurons x trajectories). No significant difference between empirical and null distributions (p = 0.37, two-sample Kolmogorov-Smirnov test).

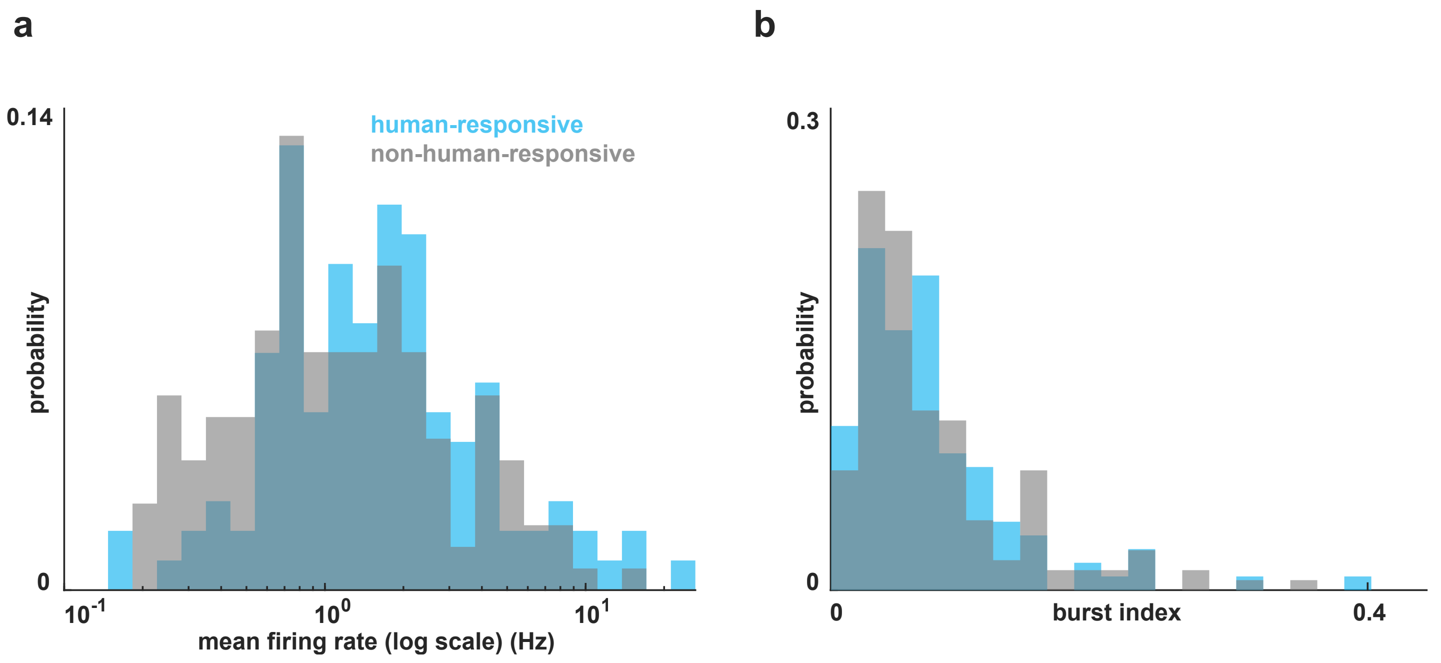

Supplementary Fig. 4 | Units properties for human-responsive v.s. non-human-responsive units.
a. Distribution of mean firing rates (log scale) of human responsive units (blue) (n = 117) and non-human-responsive units (grey) (n = 158). Significant difference between two distributions ($p = 0.02$, two-sample Kolmogorov-Smirnov test). **b.** Distribution of burst index of human responsive units and non-human-responsive units. No significant difference between the two distributions ($p = 0.47$, two-sample Kolmogorov-Smirnov test).

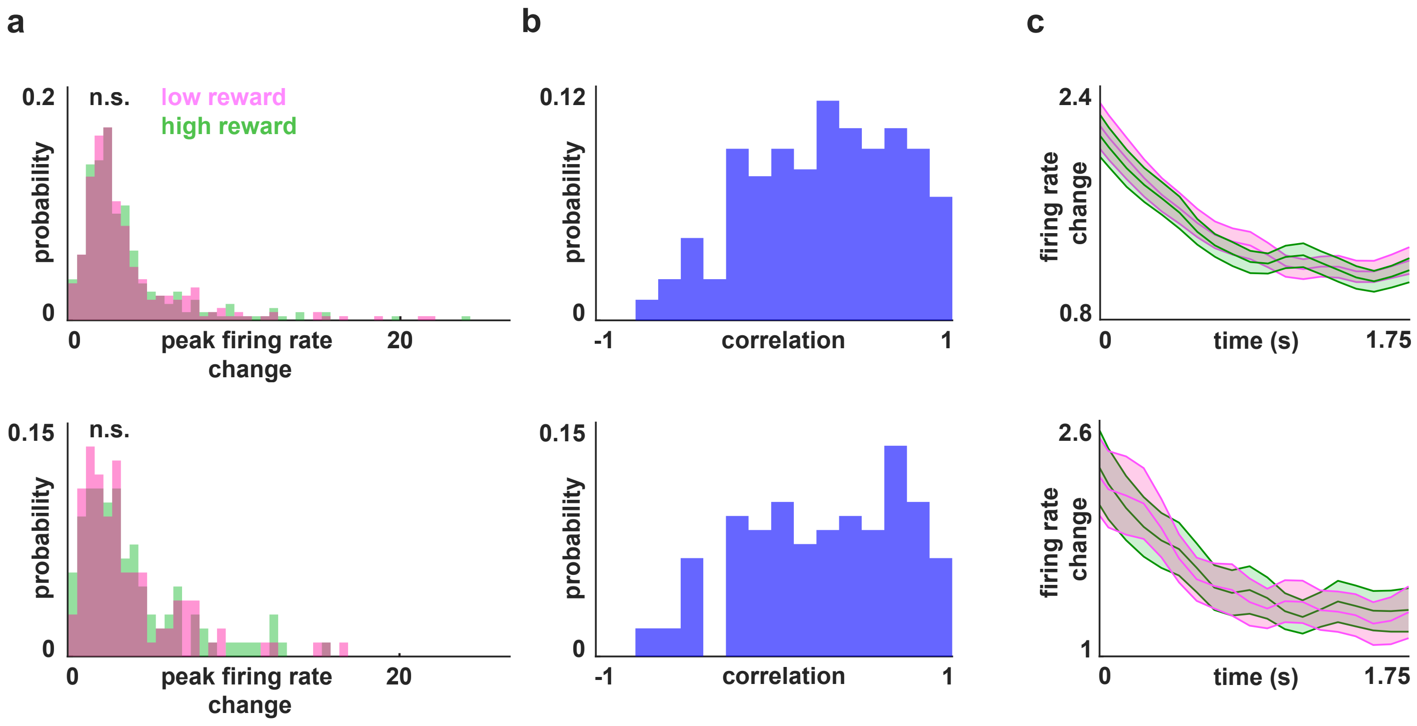

Supplementary Fig. 5 | Firing rate properties with unequal reward quantities.
a. (*top*) Distribution of peak firing rates in 2 s interval around landing for all analyzable neurons x landing locations in experiment #1, variant #2 – where different humans were stationary and provided different quantities of reward (Methods; n = 284 analyzable neurons x landing locations). No significant difference in distributions of peak firing rate change between high reward and low reward landings (p = 0.94, two-sided Wilcoxon signed rank test). (*bottom*) Same as the top but only including the neurons that were human-modulated (n = 111 neurons x landing locations). No significant difference in distributions of peak firing rate change between the different amounts of reward (p = 0.77, two-sided Wilcoxon signed rank test). **b.** (*top*) Spearman correlation between average firing rates around landing at high versus low reward locations for all analyzable neurons x landing locations, same as in subpanel '**a**'. (*bottom*) Same as the top but only including the neurons that were human-modulated, same as in the bottom of subpanel '**a**'. **c.** (*top*) Grand average firing rate changes after landing (around reward consumption) at high versus low reward locations (0 to +1.75 s, landing is 0). Shaded region indicates standard error. (*bottom*) Same as the top but only including the units that were human-modulated, same as in the bottom of subpanel '**a**'. Note that different rewards quantities by different experimenters were only provided on a subset of experiment #1 (humans are stationary while bats are flying) yet the modulation by human identity was observed irrespective of whether both humans provided different or similar reward quantities.

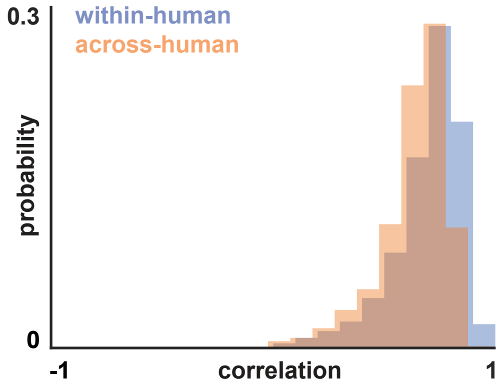

Supplementary Fig. 6 | Correlation of velocity profile for all human traverses.
Correlation of the velocity profile between all traverses of the same human (blue), and between all traverses across different humans (orange), for all sessions (n = 15 sessions; n = 5,904 traverse pairs within human, n = 5,829 traverse pairs across humans).
