# Supplementary material for: Neural representation of human experimenters in the bat hippocampus
Source: Nat Neurosci. 2024 Jul 2;27(9):1675–9. doi: 10.1038/s41593-024-01690-8 (PMC11374686; doi:10.1038/s41593-024-01690-8)
Supplement: Supplementary file 2 — Reporting Summary [file 41593_2024_1690_MOESM2_ESM.pdf]

Corresponding author(s): Michael M. Yartsev

Last updated by author(s): May 15, 2024

Reporting Summary

Nature Portfolio wishes to improve the reproducibility of the work that we publish. This form provides structure for consistency and transparency in reporting. For further information on Nature Portfolio policies, see our Editorial Policies and the Editorial Policy Checklist.

Statistics

For all statistical analyses, confirm that the following items are present in the figure legend, table legend, main text, or Methods section.

n/a	Confirmed	
☐	☒	The exact sample size (*n*) for each experimental group/condition, given as a discrete number and unit of measurement
☐	☒	A statement on whether measurements were taken from distinct samples or whether the same sample was measured repeatedly
☐	☒	The statistical test(s) used AND whether they are one- or two-sided<br>*Only common tests should be described solely by name; describe more complex techniques in the Methods section.*
☐	☒	A description of all covariates tested
☐	☒	A description of any assumptions or corrections, such as tests of normality and adjustment for multiple comparisons
☐	☒	A full description of the statistical parameters including central tendency (e.g. means) or other basic estimates (e.g. regression coefficient) AND variation (e.g. standard deviation) or associated estimates of uncertainty (e.g. confidence intervals)
☐	☒	For null hypothesis testing, the test statistic (e.g. *F*, *t*, *r*) with confidence intervals, effect sizes, degrees of freedom and *P* value noted<br>*Give P values as exact values whenever suitable.*
☒	☐	For Bayesian analysis, information on the choice of priors and Markov chain Monte Carlo settings
☒	☐	For hierarchical and complex designs, identification of the appropriate level for tests and full reporting of outcomes
☐	☒	Estimates of effect sizes (e.g. Cohen's *d*, Pearson's *r*), indicating how they were calculated

Our web collection on statistics for biologists contains articles on many of the points above.

Software and code

Policy information about availability of computer code

Data collection	Custom scripts in Python 3.9 (Ubuntu 18.04 Bionic) and Matlab 2021a were written to collect 3D positional data using an adaption of a commercially available RTLS system (Ciholas Inc.; scripts available from the corresponding author on request), and Cortex Motion Analysis software (6.2.13.1751), respectively. Neural data was acquired using a wireless neural logger (Deuteron Technologies, MouseLog16). Scripts to control reward delivery were written using Arduino programming software (ADE 1.8.19) and Python (ver 3.9). Video data was collected with the Basler Pylon software (6.2.0.8205).
Data analysis	All code for analysis was written in Matlab 2022a and is available from the corresponding author on reasonable request.

For manuscripts utilizing custom algorithms or software that are central to the research but not yet described in published literature, software must be made available to editors and reviewers. We strongly encourage code deposition in a community repository (e.g. GitHub). See the Nature Portfolio guidelines for submitting code & software for further information.

Data

Policy information about availability of data

All manuscripts must include a data availability statement. This statement should provide the following information, where applicable:

- Accession codes, unique identifiers, or web links for publicly available datasets
- A description of any restrictions on data availability
- For clinical datasets or third party data, please ensure that the statement adheres to our policy

The data presented in this study will be made available upon reasonable request.

Field-specific reporting

Please select the one below that is the best fit for your research. If you are not sure, read the appropriate sections before making your selection.

☒ Life sciences ☐ Behavioural & social sciences ☐ Ecological, evolutionary & environmental sciences

For a reference copy of the document with all sections, see nature.com/documents/nr-reporting-summary-flat.pdf

Life sciences study design

All studies must disclose on these points even when the disclosure is negative.

Sample size	We analyzed a dataset of 307 single-units recorded using wireless electrophysiology from 4 bats. No statistics were used to determine sample size. Sample sizes are similar to those used in the field (e.g. Dotson et al., Science 373.6551 (2021): 242-247. Sarel et al., Nature 609, 119–127 (2022). Forli et al., Nature 621, 796-803 (2023).
Data exclusions	The inclusion criteria for the neurons were based on sufficient behavioral coverage, spike number, and firing stability (Methods), and are standard for this research field. The inclusion criteria for behavioral sessions was sufficient behavioral coverage. Exclusion criteria were not predetermined.
Replication	Findings on neural activity and structured behavior were replicated across multiple animals, cells, and experiments. The reported effects were found across all tested animals (Methods). Experiments consisted of daily sessions during which single-units were collected (typically 4-16 per recorded bat).
Randomization	NR. Our study did not treat experimental groups and no randomization was required.
Blinding	NR. Our study did not treat experimental groups and no randomization was required.

Reporting for specific materials, systems and methods

We require information from authors about some types of materials, experimental systems and methods used in many studies. Here, indicate whether each material, system or method listed is relevant to your study. If you are not sure if a list item applies to your research, read the appropriate section before selecting a response.

Materials & experimental systems

n/a	Involved in the study	
☐	☒	Antibodies
☒	☐	Eukaryotic cell lines
☒	☐	Palaeontology and archaeology
☐	☒	Animals and other organisms
☒	☐	Human research participants
☒	☐	Clinical data
☒	☐	Dual use research of concern

Methods

n/a	Involved in the study	
☒	☐	ChIP-seq
☒	☐	Flow cytometry
☒	☐	MRI-based neuroimaging

Antibodies

Antibodies used	Goat anti-Iba1, ab5076, Abcam; Rabbit anti-PCP4, HPA005792, Sigma; Donkey anti-goat Alexa-647, Invitrogen A32849; Donkey anti-rabbit Alexa-488, Invitrogen A-21206.
Validation	Antibodies adopted in this study were from similar immunohistochemistry protocols in bats (Forli et al., Nature 621, 796-803 (2023)).

Animals and other organisms

Policy information about studies involving animals; ARRIVE guidelines recommended for reporting animal research

Laboratory animals	Four lab-born adult (3-6 years old) female Egyptian fruit bats (Rousettus aegyptiacus) were used in this study.
Wild animals	No wild-caught animals were used in this study.
Field-collected samples	No field samples were used in this study.
Ethics oversight	All procedures performed in this study were approved by the Institutional Animal Care and Use Committee at the University of California, Berkeley, and were performed in accordance with the Guide for the Care and Use of Laboratory Animals.

Note that full information on the approval of the study protocol must also be provided in the manuscript.
